# Seasonal variation in hospitalizations for peptic ulcer disease: A five-year retrospective study from Latvia

**DOI:** 10.1371/journal.pone.0345328

**Published:** 2026-03-18

**Authors:** Abdulrahman Al-Dawoudi, Mujahed Dalain, Daniil Varlamov, Nazar Kopytko, Davis Freimanis

**Affiliations:** 1 Faculty of Medicine and Life Sciences, University of Latvia, Riga, Latvia; 2 Medical Faculty No. 1, Poltava State Medical University, Poltava, Ukraine; 3 Department of Gastroenterology, Pauls Stradins Clinical University Hospital, Riga, Latvia; Jimma University, ETHIOPIA

## Abstract

Peptic ulcer disease (PUD) remains a significant global health burden, yet data on seasonal variations in PUD hospitalizations are inconsistent across regions. No studies have assessed seasonal patterns in PUD hospitalizations in Latvia or the broader Baltic region. This study aimed to evaluate whether seasonal factors influence PUD hospitalizations in this temperate-climate setting. We conducted a retrospective cohort study of adult hospitalizations with a primary discharge diagnosis of PUD (ICD-10 codes K25–K28) from 2020 to 2024 at a tertiary care hospital in Riga, Latvia. Seasonal distribution, ulcer subtype, bleeding status, length of stay (LOS), and in-hospital mortality were analyzed using chi-square, Mann–Whitney U, and Kruskal–Wallis tests, as appropriate. Multivariable logistic regression was performed to assess independent predictors of in-hospital mortality. A total of 606 hospitalizations were analyzed (median age: 66 years; 59.1% male). The median LOS was 6 days. Older adults (≥65 years) had higher in-hospital mortality compared with those <65 years (11.7% vs. 3.5%, representing an absolute difference of 8.2%). Seasonal distribution was relatively even: 22.4% in winter, 23.4% in spring, 30.4% in summer, and 23.8% in fall, with a maximum absolute difference of 8.0% between peak and trough seasons; no significant seasonal differences were observed in demographic or clinical characteristics. In multivariable analysis, season was not independently associated with in-hospital mortality (overall Wald p = 0.952), whereas increasing age was independently associated with mortality (adjusted odds ratio [aOR] 1.04 per year; 95% CI 1.02–1.07; p < 0.001). This study provides the first regional evidence from the Baltic area suggesting that seasonal climatic variation does not substantially influence severe PUD hospitalization patterns within a centralized European healthcare system.

## Introduction

Peptic ulcer disease (PUD) remains a common gastrointestinal condition worldwide, contributing substantially to morbidity and healthcare utilization [1–6]. It has an estimated lifetime prevalence of 5–10% in the general population [7,8]. PUD is associated with significant complications, such as bleeding and perforation, which affect 10–20% of patients [9]. The widespread use of nonsteroidal anti-inflammatory drugs (NSAIDs) and *Helicobacter pylori (H. pylori)* infection remain major contributors to ulcer development and gastrointestinal bleeding risk [10–12], consistent with recent global analyses confirming their continued predominance [13].

While studies from regions like South Korea and Taiwan report winter peaks [14,15], and Italy and the U.S. exhibit varying seasonal trends [16,17], it is essential to explore whether such patterns are relevant to Latvia’s unique climate and healthcare practices. Data from Norway show high rates of NSAID use among patients with ulcer bleeding [18]. Proposed mechanisms for this variation include the increased use of NSAIDs in colder months, which may contribute to seasonal variation in PUD hospitalizations. This is compounded by impaired gastric mucosal protection due to cold weather and higher alcohol consumption [19–22].

While seasonal patterns have been highlighted in various regions, they have not been explored in Latvia or the broader Baltic region, which experiences harsh winters and distinct climatic conditions. Building on this gap in the literature, this study aims to evaluate whether seasonal factors influence PUD hospitalizations in Latvia, providing new insights into the seasonal dynamics of the disease in this unique setting.

The primary objective of this study was to assess whether hospitalizations for peptic ulcer disease (PUD) at a tertiary care hospital in Riga, Latvia, demonstrated seasonal variation over a five-year period (2020–2024). Secondary objectives included characterizing the demographic and clinical features of hospitalized PUD patients—including age, sex, ulcer subtype, bleeding status, length of stay, and in-hospital mortality—and investigating whether these factors were associated with differences in clinical outcomes. Additionally, the study aimed to compare the seasonal distribution of PUD hospitalizations in Latvia with findings from regions with similar temperate climates, to contextualize potential environmental influences on disease patterns.

## Materials and methods

### Study design and setting

This single-center, retrospective cohort study was conducted at Pauls Stradins Clinical University Hospital (PSCUH) in Riga, Latvia. The database used for this study captures hospitalizations exclusively at PSCUH and does not include data from other public or private hospitals in Latvia. PSCUH is a large tertiary academic referral center, providing emergency and specialized care to the Riga metropolitan region and receiving referrals from other areas of Latvia. As one of the largest and most comprehensive hospitals in Latvia, PSCUH primarily reflects the clinical course of more severe PUD cases requiring hospitalization. However, the study may not fully represent the entire spectrum of PUD cases in Latvia, as milder cases treated in primary care settings or outpatient facilities were not included. Therefore, while the findings are applicable to severe PUD hospitalizations, they may not fully reflect less severe cases that do not require hospitalization.

Hospital discharge diagnoses are recorded using the International Classification of Diseases, 10th Revision (ICD-10) within a centralized electronic medical record system. Diagnostic codes are entered by treating physicians and processed through the hospital’s standardized coding and reporting system in accordance with national administrative requirements. Internal quality control procedures, such as periodic physician training and auditing of randomly selected patient records, are routinely applied to ensure the completeness and consistency of discharge data. However, independent external validation of diagnostic coding was not performed, and the possibility of misclassification cannot be entirely excluded.

During the study period (2020–2024), no major changes in ICD-10 coding standards or institutional diagnostic coding policies occurred at PSCUH. However, the early phase of the COVID-19 pandemic (2020–2021) may have influenced healthcare utilization patterns, including shifts in hospital admission rates and changes in treatment protocols. To address this potential influence, a sensitivity analysis incorporating a binary COVID-period indicator (2020–2021 vs. 2022–2024) was conducted.

### Study population and eligibility criteria

Hospital records were screened to identify all adult hospitalizations (≥ 18 years) between January 1, 2020, and December 31, 2024, with a primary discharge diagnosis of peptic ulcer disease (PUD), defined by ICD-10 codes K25–K28 (gastric ulcer, duodenal ulcer, peptic ulcer unspecified, and gastrojejunal ulcer).

Exclusion criteria were:

- Rehospitalizations for PUD occurring within 30 days were excluded; only the first (index) hospitalization was included.
- Records with missing or incomplete key variables.
- Outpatient cases not requiring hospitalization.
- Cases in which K25–K28 codes were not recorded as the primary discharge diagnosis.

While ulcer subtypes were categorized and analyzed separately in descriptive and subgroup analyses, the primary seasonal analysis evaluated overall PUD hospitalizations to assess aggregate seasonal patterns.

### Variable definitions

The season of admission was defined according to standard meteorological definitions (winter: December–February; spring: March–May; summer: June–August; fall: September–November). This classification is widely used in epidemiological studies, particularly in temperate climates, as it aligns with natural climatic cycles and provides uniform, three-month intervals—both essential for accurately assessing seasonal variation in disease incidence. This approach has been consistently applied across various regions, ensuring comparability with international research on seasonal trends in health outcomes. For instance, studies by Manfredini et al. (2010) in Italy [16] and Yoon et al. (2021) in Korea [14] applied standard meteorological seasonal classification to evaluate patterns in peptic ulcer disease and related complications like peptic ulcer bleeding (PUB). By using this widely accepted methodology, our study aims to provide a standardized framework for evaluating seasonal variation in peptic ulcer disease that is consistent with the global body of epidemiological literature.

Ulcer subtype was classified according to the primary ICD-10 diagnosis:

- Gastric ulcer (K25)
- Duodenal ulcer (K26)
- Unspecified peptic ulcer (K27)
- Gastrojejunal ulcer (K28)

Ulcer subtypes were analyzed together in the primary analysis due to their similar pathophysiological mechanisms and clinical management, although separate subgroup analyses were performed to assess potential differences in outcomes.

Bleeding status was determined from the ICD-10 primary discharge code. Codes indicating acute ulcer with hemorrhage (e.g., K25.0, K25.2, K26.0, K26.2) were classified as bleeding ulcers; all other K25–K28 codes were categorized as non-bleeding. This classification helps distinguish the clinical severity, as bleeding ulcers typically require more intensive management and are associated with worse outcomes.

Length of stay (LOS) was calculated using the admission and discharge calendar dates as (discharge date − admission date) + 1, such that same-day hospitalizations were coded as 1 day.

Age was analyzed as a continuous variable and dichotomized into <65 years and ≥65 years for subgroup comparisons. This categorization was based on clinical considerations and prior literature indicating that age ≥ 65 years is a significant risk factor for worse outcomes in peptic ulcer disease.

### Outcomes

**Primary outcome:**

- Seasonal variation in PUD hospitalizations.

**Secondary outcomes:**

- Ulcer subtype distribution
- Frequency of bleeding vs non-bleeding ulcers
- Length of stay
- In-hospital mortality (including adjusted analyses)
- Age-related and gender-related differences in outcomes

### Ethical considerations

The study was approved by the Medical Research Ethics Committee of the Faculty of Medicine and Life Sciences, University of Latvia (approval No. 23–37/269). The requirement for informed consent was waived by the ethics committee due to the retrospective cohort design and the use of routinely collected administrative data without direct patient contact. All procedures were conducted in accordance with the Declaration of Helsinki and applicable national regulations governing biomedical research.

Data extraction was performed by the hospital IT department within the secure institutional electronic medical record system. The research team had access to coded data during the data preparation phase; however, no direct personal identifiers (including names, personal identification numbers, or contact information) were accessible at any time. All data were anonymized prior to statistical analysis, and all analyses were conducted using fully de-identified datasets. The anonymized dataset was accessed for research purposes on 13 November 2025.

### Statistical analysis

The statistical analysis was performed using IBM SPSS Statistics version 29 (IBM Corp., Armonk, NY). A two-sided p-value < 0.05 was considered statistically significant for all analyses.

### Descriptive statistics

Categorical variables were summarized as frequencies and percentages, while continuous variables, which were found to be non-normally distributed, were summarized as medians with interquartile ranges (IQR). In subgroup comparison tables, the interquartile range is presented as IQR width (calculated as Q3 − Q1) for clarity and consistency.

### Comparison of groups

Group comparisons for categorical variables were performed using chi-square tests. For continuous variables, comparisons between two groups were made using the Mann–Whitney U test, and comparisons across multiple groups were performed using the Kruskal–Wallis test. Seasonal differences in hospitalization frequency were assessed using a χ^²^ goodness-of-fit test. Because the primary objective was to evaluate relative seasonal distribution rather than model continuous month-to-month temporal trends, categorical seasonal comparison using χ^²^ testing was considered methodologically appropriate and statistically justified for the present analysis in this study.

Because the primary objective was to evaluate differences in the distribution of hospitalizations across predefined seasons, an a priori χ^²^ goodness-of-fit power analysis (G*Power 3.1; df = 3; α = 0.05) was conducted to ensure adequate statistical power. This analysis indicated that a minimum sample size of 549 hospitalizations would be required to detect a 10 percentage-point difference between seasons with 80% power. The available sample (N = 606) exceeded this threshold and therefore provided sufficient statistical power for the primary seasonal comparison.

### Multivariable analysis and sensitivity analysis

A multivariable logistic regression model was applied to identify independent predictors of in-hospital mortality (dead vs. transferred/discharged). The model included the following covariates: age (continuous), sex (categorical), year of hospitalization (continuous) to account for temporal trends in healthcare utilization, and season of admission (categorical) to assess any seasonal effects. Season (winter as the reference category) and sex (female as the reference category) were entered as categorical variables, while age and year of hospitalization were modeled as continuous covariates. However, season was not independently associated with mortality (overall Wald test p = 0.952). The inclusion of age and sex was based on established risk factors for PUD mortality, and no evidence of problematic multicollinearity was identified among included covariates. For each predictor, adjusted odds ratios (aOR) with 95% confidence intervals (CI) were calculated to assess the strength of the association.

To assess the potential impact of the COVID-19 pandemic (2020–2021) on hospitalization rates and in-hospital mortality, a sensitivity analysis was conducted by replacing the hospitalization year covariate with a binary COVID-period indicator (2020–2021 vs. 2022–2024). Adjusted odds ratios (aOR) for in-hospital mortality were calculated for the COVID and post-COVID periods to evaluate any residual impact of the pandemic on patient outcomes.

## Results

**Fig 1:**
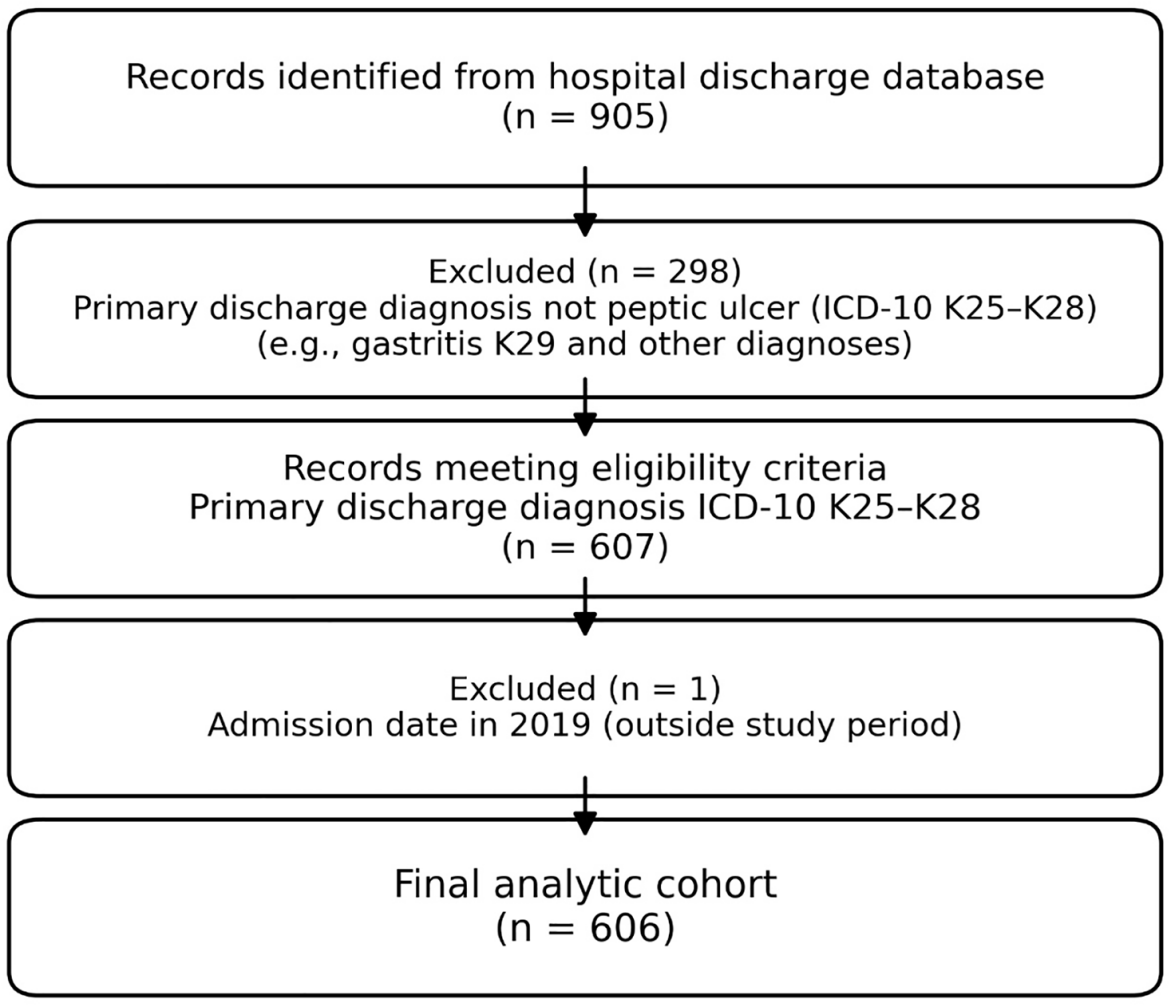
Cohort selection process. Hospital records were screened using ICD-10 codes K25–K28, identifying 905 encounters. After applying eligibility and exclusion criteria, 606 unique patient hospitalizations were included in the final analytic cohort.

The cohort selection process involved screening hospital records using ICD-10 codes K25–K28, which initially identified 905 encounters. After applying eligibility and exclusion criteria, 606 unique patient hospitalizations were included in the final analytic cohort, as detailed in Fig 1.

Among the 606 hospitalizations for peptic ulcer disease (PUD) between 2020 and 2024, the median age was 66 years (IQR 53.75–78), and 59.1% were male. Among the ulcer subtypes, 63.4% were diagnosed with gastric ulcers, followed by duodenal ulcers at 34.0%. Of these patients, 587 (96.9%) were classified as having bleeding ulcers, while 19 (3.1%) were identified as having non-bleeding ulcers. The characteristics of these patients are summarized in Table 1.

**Table 1:** Baseline characteristics of the study population (N = 606).

Variable	Category/ Statistic	Value
Year of hospitalization	2020	122 (20.1)
	2021	114 (18.8)
	2022	102 (16.8)
	2023	130 (21.5)
	2024	138 (22.8)
Sex	Female	248 (40.9)
	Male	358 (59.1)
Discharge status	Discharged home	537 (88.6)
	Died	48 (7.9)
	Transferred	21 (3.5)
Ulcer subtype	Gastric ulcer (K25)	384 (63.4)
	Duodenal ulcer (K26)	206 (34.0)
	Peptic ulcer, unspecified (K27)	10 (1.7)
	Gastrojejunal ulcer (K28)	6 (1.0)
Bleeding status	Bleeding	587 (96.9)
	Non-bleeding	19 (3.1)
Season of admission	Winter	136 (22.4)
	Spring	142 (23.4)
	Summer	184 (30.4)
	Fall	144 (23.8)

Values are presented as n (%) of the total study population (N = 606). K25–K28 represent International Classification of Diseases, 10th Revision (ICD-10) diagnostic codes.

**Fig 2:**
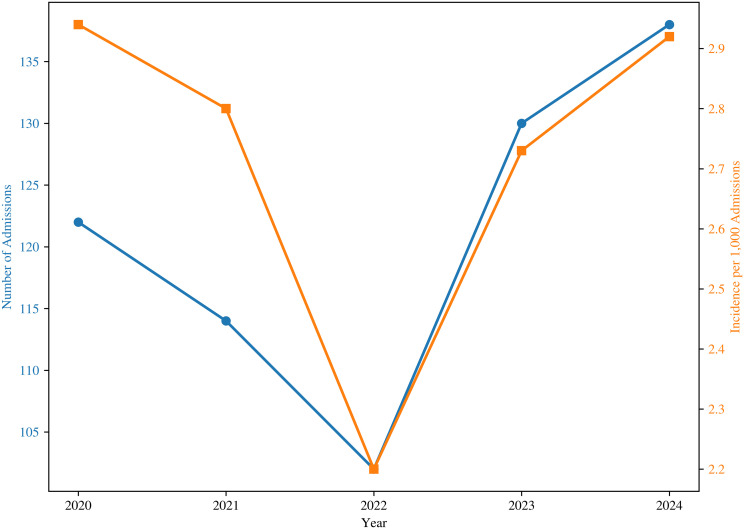
Annual hospitalizations for peptic ulcer disease (2020–2024). Yearly variation in PUD hospitalizations at a tertiary care hospital in Riga, ranging from 102 to 138 cases, with incidence per 1,000 adult hospitalizations shown for each year.

**Fig 3:**
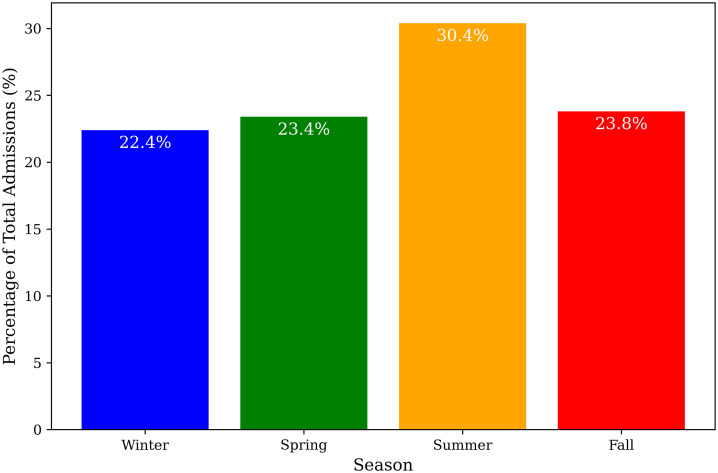
Seasonal distribution of peptic ulcer disease hospitalizations. PUD hospitalizations across the four seasons, showing a relatively even distribution without significant seasonal differences in demographic or clinical characteristics.

Annual PUD case counts ranged from 102 to 138, with the lowest frequency in 2022 and the highest in 2024 (Fig 2). The annual incidence varied from 2.20 to 2.94 cases per 1,000 adult hospitalizations. Seasonal distribution was relatively uniform, with 30.4% of cases occurring in summer and 22.4% in winter, representing a maximum absolute difference of 8.0% between peak and trough seasons (Fig 3). Seasonal comparisons did not demonstrate meaningful differences in patient age (Kruskal–Wallis p = 0.052), sex distribution (χ² p = 0.770), ulcer subtype (χ² p = 0.652), length of stay (Kruskal–Wallis p = 0.098), or unadjusted in-hospital mortality (χ² p = 0.900); detailed seasonal comparisons are provided in Table 2. Older adults (≥65 years) had higher unadjusted in-hospital mortality compared with those <65 years (11.7% vs. 3.5%, representing an absolute difference of 8.2%; p < 0.001).

**Table 2:** Seasonal comparison of demographic and clinical characteristics in peptic ulcer disease hospitalizations.

Variable	Winter (n = 136)	Spring (n = 142)	Summer (n = 184)	Fall (n = 144)	p-value
Age, median (IQR width), years	69 (22)	65.5 (24)	64 (28)	65.5 (23)	0.052^a^
Male, n (%)	75 (55.1)	85 (59.9)	111 (60.3)	87 (60.4)	0.770^b^
LOS, median (IQR width), days	6 (4)	7 (4)	6 (5)	7 (5)	0.098^a^
Ulcer subtype, n (%)					
Gastric ulcer	82 (60.3)	91 (64.1)	116 (63.0)	95 (66.0)	0.652^b^
Duodenal ulcer	52 (38.2)	47 (33.1)	65 (35.3)	42 (29.2)	
Unspecified ulcer	1 (0.7)	3 (2.1)	2 (1.1)	4 (2.8)	
Gastrojejunal ulcer	1 (0.7)	1 (0.7)	1 (0.5)	3 (2.1)	
In-hospital mortality, n (%)	13 (9.6)	11 (7.7)	14 (7.6)	10 (6.9)	0.900^b^
Transferred, n (%)	4 (2.9)	3 (2.1)	8 (4.3)	6 (4.2)	

^a^Kruskal–Wallis test.

^b^Chi-square test.

Values presented as median (IQR width, calculated as Q3 − Q1) or n (% within season). IQR, interquartile range; LOS, length of stay.

In the multivariable logistic regression model adjusted for age, sex, and hospitalization year, season was not independently associated with in-hospital mortality (overall Wald p = 0.952).

Compared with winter, the adjusted odds ratios (aOR) were 0.93 (95% CI 0.39–2.21) for spring, 0.92 (95% CI 0.41–2.06) for summer, and 0.77 (95% CI 0.32–1.86) for fall. Age was independently associated with in-hospital mortality (aOR 1.04 per year increase, 95% CI 1.02–1.07; p < 0.001), whereas sex was not independently associated with mortality after adjustment (aOR 0.60, 95% CI 0.31–1.16; p = 0.127). These results are summarized in Table 3.

**Table 3:** Multivariable logistic regression analysis of factors associated with in-hospital mortality.

Variable	Adjusted Odds Ratio (aOR)	95% CI	p-value
Age (per year increase)	1.04	1.02–1.07	< 0.001
Sex (reference = Female)			
Male	0.60	0.31–1.16	0.127
Season of admission			0.952^a^
Season of admission (reference = Winter)			
Spring	0.93	0.39–2.21	0.873
Summer	0.92	0.41–2.06	0.834
Fall	0.77	0.32–1.86	0.567
Year of admission (per year)	0.99	0.81–1.22	0.942

^a^Overall Wald test for season variable.

Model adjusted for age, sex, year of admission, and season.

Reference categories: Female sex and Winter season.

**Fig 4:**
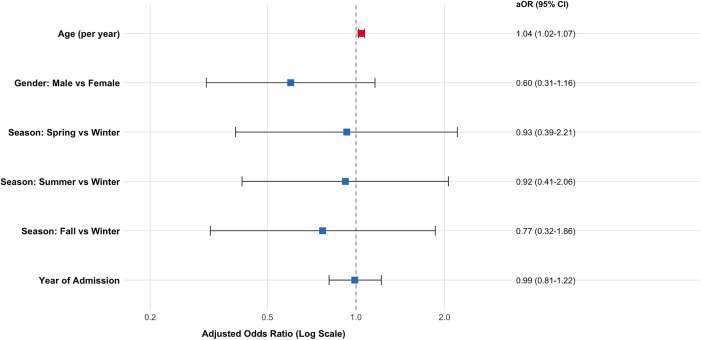
Forest plot of adjusted odds ratios (aORs) for in-hospital mortality. Multivariable logistic regression adjusted for age (per year), sex (reference = female), year of admission (per year), and season of admission (reference = winter). Error bars indicate 95% confidence intervals. Odds ratios are presented on a logarithmic scale.

Findings were consistent in a sensitivity analysis replacing hospitalization year with a binary COVID-era indicator (2020–2021 vs. 2022–2024), which confirmed no association between season and mortality (overall Wald p = 0.949) and no significant effect of the post-COVID period (aOR 0.92, 95% CI 0.50–1.71; p = 0.790). A forest plot representation of these results is provided in Fig 4.

The median length of stay for the entire cohort was 6 days (IQR 5–9), with a mean of 7.55 ± 5.01 days. Full summaries of continuous variables are provided in S1 Table.

Annual incidence values for the five-year period are shown in Table 4.

**Table 4:** Annual incidence of peptic ulcer disease (2020–2024).

Year	PUD cases (n)	Total adult hospitalizations (n)	Incidence per 1,000 adult hospitalizations
2020	122	41,571	2.94
2021	114	40,733	2.80
2022	102	46,318	2.20
2023	130	47,590	2.73
2024	138	47,263	2.92

PUD, peptic ulcer disease.

**Fig 5:**
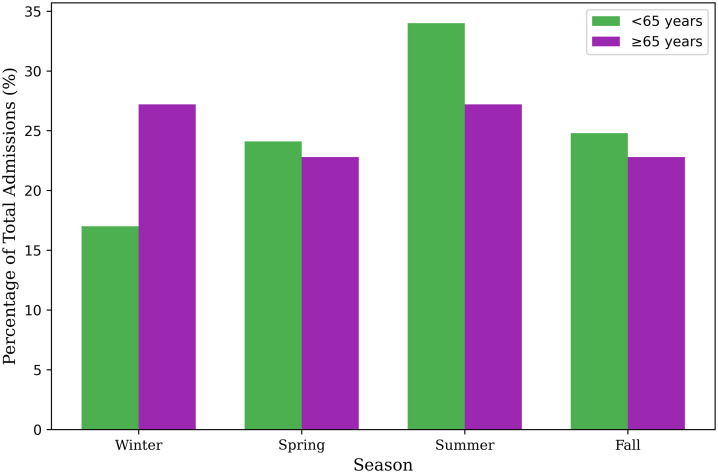
Seasonal distribution by age group. Seasonal hospitalization patterns for patients <65 years and ≥65 years, illustrating a higher proportion of winter admissions among older adults and more frequent summer admissions among younger adults.

Patients aged ≥65 years accounted for 53.5% of all hospitalizations. Seasonal distribution differed between age groups (χ² test, p = 0.022), with older adults more commonly admitted in winter (27.2% vs. 17.0%, an absolute difference of 10.2%) and younger adults more frequently admitted in summer (34.0% vs. 27.2%) (Fig 5).

Older patients also had significantly longer hospital stays and higher in-hospital mortality compared with younger adults (11.7% vs. 3.5%, representing an absolute difference of 8.2%; p < 0.001). These findings are summarized in Table 5.

**Table 5:** Comparison of patients <65 years vs ≥ 65 years.

Variable	<65 years (n = 282)	≥65 years (n = 324)	p-value
Season of admission (%)			0.022^a^
Winter	17.0	27.2	
Spring	24.1	22.8	
Summer	34.0	27.2	
Fall	24.8	22.8	
LOS, mean rank	268.42	334.03	<0.001^b^
In-hospital mortality, n (%)	3.5 (10/282)	11.7 (38/324)	<0.001^b^

^a^Chi-square test.

^b^Mann–Whitney U test.

Female patients were significantly older than male patients (p < 0.001). Length of stay did not differ significantly between sexes (p = 0.298). In-hospital mortality was higher among women compared with men (12.1% vs. 5.0%), corresponding to a 7.1% absolute difference. These findings are detailed in S2 Table.

Ulcer subtype showed significant differences in age distribution (Kruskal–Wallis H = 16.077, p = 0.001), with duodenal ulcers occurring in younger patients and unspecified or gastrojejunal ulcers occurring in older patients. However, ulcer subtype was not associated with length of stay, mortality, or seasonal pattern. Age-group distribution by subtype also differed significantly (χ²(3) = 17.689, p = 0.001), as shown in S3 Table.

## Discussion

In this five-year cohort of 606 hospitalized peptic ulcer patients, we found no statistically significant seasonal variation in hospitalization rates or clinical outcomes. This finding contrasts with several prior studies that have identified clear seasonal patterns. For example, Fares et al. systematically reviewed global data and reported that peptic ulcer disease (PUD) incidence tends to peak during colder months, suggesting a reproducible winter predominance across diverse geographic settings [23]. Likewise, Østensen et al. observed a seasonal trend in northern Norway, with higher rates of first-time gastric and duodenal ulcers from September to January; however, the variation was relatively small [24].

Large population-based analyses from Asia describe seasonal variation, with a South Korean nationwide cohort showing the highest PUD incidence in winter and the lowest in fall [14], and the Taiwanese national dataset showing that PUD incidence was highest in winter, followed by spring, with the lowest incidence in fall and summer [15]. In Europe, Manfredini et al. reported significant PUD hospitalization peaks in late summer–fall and winter, with a July nadir [16].

North American data are mixed; Kanotra et al. (US) found spring peaks and fall troughs in PUD hospitalizations [17], whereas a recent US analysis by Yaratha et al. reported the highest hospitalizations in fall and the lowest in summer [25]. Thus, while some temperate-region studies report fall/winter surges [14,15,23,24], others show varying patterns depending on the region and time period.

Although Latvia experiences long, cold winters, we observed no statistically significant seasonal differences in hospitalizations or outcomes. This finding remained consistent across multivariable and COVID-era sensitivity analyses.

Seasonal factors appear to play only a limited role in hospitalization patterns in this setting. Patient-level risk factors, with age being a primary example, should therefore remain the focus of prevention and risk stratification strategies. These results offer important insights into the relationship between climate, healthcare practices, and disease management in the Baltic region, which may differ from patterns established in other temperate climates. This finding is consistent with data from Spain reporting no significant monthly, seasonal, or climatic variation in hospitalizations for upper gastrointestinal bleeding, including cases due to gastric and duodenal ulcers [26].

Previous studies reporting seasonal variation in PUD hospitalizations have proposed that fluctuations in NSAID exposure, behavioral stressors, and environmental factors may contribute to the temporal clustering of acute presentations [15,17,27].

The absence of a statistically significant seasonal pattern in Latvia may reflect specific regional structural and healthcare system characteristics. Latvia operates a publicly funded, centralized healthcare system in which residents have consistent year-round access to primary and tertiary care [28]. Emergency endoscopy services are available 24/7 at our tertiary center, and both NSAIDs and proton pump inhibitors are readily accessible throughout the year [29]. Within this nationally coordinated framework, healthcare delivery, prescribing practices, and hospital admission thresholds remain stable across seasons [30]. In Latvia, NSAIDs and gastroprotective agents are widely available year-round through regulated pharmacy distribution, and there is no documented seasonal restriction or access variability that would be expected to generate cyclical exposure patterns.

Although the Baltic region experiences pronounced climatic variation, including prolonged winters and substantial temperature fluctuations, the structural stability of healthcare access and medication availability may attenuate observable seasonal clustering of severe PUD hospitalizations. While speculative, these structural characteristics may partially contribute to the relatively uniform hospitalization patterns observed.

In contrast, some Asian healthcare systems, where winter predominance in ulcer-related complications has been reported [14,15], may experience greater seasonal variability in healthcare utilization, outpatient access, medication adherence, or elective endoscopic availability. These differences may amplify winter clustering of ulcer-related complications.

Differences in prescribing practices, over-the-counter medication regulation, *H. pylori* epidemiology, and hospital triage policies may further contribute to the divergence in observed seasonal patterns across regions. These structural healthcare differences may help explain why Latvia does not exhibit the winter predominance observed in several Asian cohorts.

Given the lack of seasonal variation, we next evaluated whether patient-level characteristics influenced outcomes. Age was a strong prognostic determinant, with older patients experiencing markedly worse outcomes, consistent with prior literature [31–34]. In our cohort, patients aged ≥65 years had significantly longer hospital stays and higher in-hospital mortality (11.7% vs. 3.5% among those <65 years). This pattern aligns with established evidence, such as that reported by Christensen et al., who found that 30-day mortality after bleeding peptic ulcer disease was 4.3% in patients younger than 65 years, increasing to 16.9% in those aged 80 years or older [26]. Advanced age remained a strong independent risk factor in those analyses, even after adjusting for comorbidities. Similarly, global burden-of-disease data emphasize that the elderly bear a disproportionate PUD burden, with complications such as bleeding and perforation being more common in this demographic [35].

Regarding sex differences, women in our cohort had higher in-hospital mortality (12.1% vs. 5.0%). However, this excess mortality is more plausibly explained by their significantly older age at presentation rather than a true sex-specific biological effect. Previous population-based studies have consistently shown that female patients with complicated peptic ulcer disease tend to present at older ages than men. In a Norwegian cohort of perforated peptic ulcer, women were significantly older (median 73 vs. 62 years), and mortality increased markedly with age, while no independent mortality difference between sexes was observed after age adjustment [34]. Similarly, historical U.S. epidemiologic data demonstrated that narrowing sex differences in ulcer-related hospitalization and mortality were largely attributable to increasing rates among older women rather than intrinsic biological susceptibility [36]. Other hospital-based analyses have likewise reported that women with ulcer-related admissions are older than men and that age remains the dominant prognostic determinant [16]. A Swedish cohort study of patients hospitalized with peptic ulcer bleeding likewise demonstrated that age and comorbidity burden were the principal independent predictors of mortality, whereas sex was not independently associated with in-hospital death after multivariable adjustment [37].

Taken together, these findings suggest that age and comorbidity burden—rather than biological sex per se—are the primary drivers of mortality in hospitalized peptic ulcer populations. In our cohort, the significantly older age of female patients likely accounts for the observed mortality difference. Nonetheless, because detailed comorbidity and medication data were unavailable, residual confounding cannot be excluded, and future multicenter studies with comprehensive risk adjustment are warranted to clarify whether any independent sex-based effect persists.

Ulcer characteristics appeared to have little impact on outcomes. The vast majority (96.9%) were classified as bleeding ulcers, which reflects the nature of hospitalized PUD cases that are typically more severe and complicated. This aligns with large-database studies, where the majority of PUD hospitalizations involve hemorrhage or perforation [25]. The high rate of bleeding ulcer cases in our cohort may have homogenized the sample, potentially obscuring any seasonal trends that could have been observed in non-bleeding cases.

We found no meaningful differences in length of stay or discharge outcomes between duodenal and gastric ulcers. Our cohort was strongly gastric-predominant, with nearly two-thirds of ulcers classified as gastric. This distribution is consistent with reports indicating that demographic shifts, declining *H. pylori* prevalence, and widespread NSAID/aspirin use have contributed to changes in the epidemiology of peptic ulcer disease [7,13]. Importantly, regional differences in *H. pylori* prevalence may also influence observed seasonal patterns. Population-based data from Latvia indicate *H. pylori* seroprevalence of 79.2% [38], comparable to rates reported in many Asian populations where prevalence often exceeds 80% [39].

Yet, despite similarly high infection rates, we observed no seasonal variation in PUD hospitalizations. These findings suggest that *H. pylori* prevalence alone does not determine seasonal patterns. Rather, the dominant mechanism of ulcer pathogenesis may be more critical. In Latvia’s aging population, where NSAID/antiplatelet use is widespread and *H. pylori*-attributable ulcers have declined [7,13], ulcer risk may be driven by continuous medication exposure rather than infection-related seasonal fluctuations. In contrast, in Asian populations where infection-driven pathogenesis remains predominant, seasonal immune or mucosal changes may amplify winter ulcer risk [39]. Contemporary European data demonstrate a marked reduction in *H. pylori*-attributable ulcers compared with previous decades [7,13], which may partially contribute to the absence of a pronounced seasonal signal in our Latvian cohort.

Importantly, despite differences in subtype prevalence, clinical outcomes in our data did not differ significantly between gastric and duodenal ulcer groups. This aligns with a Danish nationwide cohort study, which found that after adjusting for age, comorbidities, and other factors, ulcer location (gastric vs. duodenal) did not independently predict 30- or 90-day mortality or need for re-intervention in patients with perforated peptic ulcer disease [40].

However, the published literature remains limited, and many available studies do not stratify outcomes by ulcer subtype, which constrains direct comparison with our findings. This highlights the need for further research that differentiates the impact of ulcer type on clinical outcomes, especially in relation to comorbidities and demographic factors.

This study has several limitations. First, the single-center design limits generalizability, as the cohort primarily reflects more severe, hospitalized PUD cases managed at a tertiary referral center and may underrepresent milder presentations treated in outpatient settings. Consequently, the findings are most directly applicable to complicated PUD requiring hospitalization rather than the full clinical spectrum of ulcer disease in the general population.

Second, reliance on administrative ICD-10 discharge coding restricted access to detailed individual-level clinical data, including *H. pylori* status, NSAID or antiplatelet use, alcohol consumption, comorbidity burden, and other behavioral or socioeconomic factors. These variables are well-recognized determinants of ulcer development and complication risk and could theoretically vary across seasons. While multivariable adjustments were performed for age, sex, and year of admission, additional covariates were unavailable, and residual confounding cannot be excluded. Because our primary analysis examined relative seasonal distribution rather than absolute incidence, substantial and consistently seasonal variation in these unmeasured factors would be required to mask a clinically meaningful seasonal signal. To our knowledge, no national epidemiologic data suggest pronounced seasonal fluctuations in NSAID prescribing or healthcare access in Latvia that would plausibly obscure a strong seasonal effect.

The consistency of findings across multivariable and sensitivity analyses suggests that any such confounding is unlikely to have substantially altered the principal conclusion of minimal seasonal influence in this hospitalized cohort.

Third, the study period (2020–2024) overlapped with the COVID-19 pandemic, which may have affected healthcare-seeking behavior, hospital admission thresholds, and endoscopic service utilization, particularly during 2020–2021. Although sensitivity analyses incorporating a COVID-period indicator were performed and yielded consistent results, the precise magnitude of pandemic-related disruption cannot be fully quantified.

Extrapolation of these findings to other countries should be undertaken cautiously. Climatic variability, healthcare system organization, prescribing practices for NSAIDs and gastroprotective agents, and regional differences in H. pylori epidemiology may influence hospitalization dynamics and seasonal patterns. Thus, the absence of a pronounced seasonal signal in Latvia does not preclude different patterns in healthcare systems with alternative structural or epidemiologic profiles.

As an observational retrospective analysis, causal inferences cannot be established. Nonetheless, the use of multi-year, systematically coded hospital data provides a robust regional epidemiologic assessment and establishes an important reference framework for the Baltic context. Future multicenter studies across Northern and Eastern Europe would enhance external validity and allow cross-country comparisons. Prospective investigations incorporating detailed individual-level data on infection status, medication exposure, comorbidity burden, and socioeconomic determinants would further clarify the interaction between environmental and patient-level risk factors. Long-term time-series analyses may further refine these findings and help determine whether subtle cyclical trends emerge over extended observation periods. From a clinical and health-system perspective, the absence of strong seasonal variation suggests that preventive strategies and resource planning in Latvia may be more effectively directed toward high-risk patient characteristics—particularly advanced age and comorbidity—rather than season-based adjustments in service allocation.

## Conclusions

In this retrospective single-center cohort of hospitalized patients with peptic ulcer disease in Latvia, no statistically significant seasonal variation in admissions or in-hospital mortality was observed. Although crude mortality was higher among female patients, this difference was most likely attributable to older age at presentation rather than an independent sex-specific effect.

These findings suggest that within a centralized European healthcare system with stable year-round access to care and medications, seasonal fluctuations may play only a limited role in shaping hospitalization patterns for severe peptic ulcer disease.

Clinically, this supports the prioritization of established risk factors—such as advanced age and comorbidity burden—over seasonal considerations in risk stratification and resource planning.

Future multicenter studies incorporating detailed individual-level data on Helicobacter pylori status, NSAID exposure, comorbidities, and socioeconomic factors are warranted to further clarify potential regional differences and to determine whether subtle seasonal or sex-specific effects emerge after comprehensive risk adjustment.

## Supporting information

S1 Table Continuous variables: age and length of stay. Summary statistics and nonparametric comparisons of age and length of hospital stay between age groups.
(DOCX)

S2 Table Gender differences in clinical characteristics. Comparison of demographic and clinical variables between male and female patients.
(DOCX)

S3 Table Age distribution by ulcer subtype in peptic ulcer disease patients. Comparison of age distribution across gastric, duodenal, unspecified, and gastrojejunal ulcer subtypes, including nonparametric and chi-square analyses.
(DOCX)

S1 Data Anonymized dataset for the study. De-identified dataset used for statistical analyses.
(XLSX)
