# Supplementary material for: Seasonal variation in hospitalizations for peptic ulcer disease: A five-year retrospective study from Latvia
Source: PLoS One. 2026 Mar 18;21(3):e0345328. doi: 10.1371/journal.pone.0345328 (PMC12998839; doi:10.1371/journal.pone.0345328)
Supplement: S1 Table — Summary statistics and nonparametric comparisons of age and length of hospital stay between age groups. (DOCX) [file pone.0345328.s001.docx]

**S1 Table. Continuous variables: age and length of stay.**

| **Variable** | **Mean ± SD** | **Median** | **IQR** | **Range** |
| --- | --- | --- | --- | --- |
| Age (years) | 64.78 ± 17.20 | 66 | 53.75–78 | 18–100 |
| Length of stay (days) | 7.55 ± 5.01 | 6 | 5–9 | 1–53 |

Abbreviations: IQR, interquartile range; SD, standard deviation.
