# Supplementary material for: Seasonal variation in hospitalizations for peptic ulcer disease: A five-year retrospective study from Latvia
Source: PLoS One. 2026 Mar 18;21(3):e0345328. doi: 10.1371/journal.pone.0345328 (PMC12998839; doi:10.1371/journal.pone.0345328)
Supplement: S2 Table — Comparison of demographic and clinical variables between male and female patients. (DOCX) [file pone.0345328.s002.docx]

**S2 Table. Gender differences in clinical characteristics.**

| **Variable** | **Female (n=248)** | **Male (n=358)** | **p-value** |
| --- | --- | --- | --- |
| In-hospital mortality, n (%) | 30 (12.1) | 18 (5.0) | 0.005^a^ |
| Length of stay (mean rank) | 312.33 | 297.38 | 0.298^b^ |
| Age (mean rank) | 379.86 | 250.60 | < 0.001^b^ |

^a^Chi-square test.

^b^Mann–Whitney U test.
