# Supplementary material for: Seasonal variation in hospitalizations for peptic ulcer disease: A five-year retrospective study from Latvia
Source: PLoS One. 2026 Mar 18;21(3):e0345328. doi: 10.1371/journal.pone.0345328 (PMC12998839; doi:10.1371/journal.pone.0345328)
Supplement: S3 Table — Comparison of age distribution across gastric, duodenal, unspecified, and gastrojejunal ulcer subtypes, including nonparametric and chi-square analyses. (DOCX) [file pone.0345328.s003.docx]

**S3 Table. Age distribution by ulcer subtype in peptic ulcer disease patients.**

| **Ulcer subtype** | **n** | **Mean age rank** | **<65 years (%)** | **≥65 years (%)** |
| --- | --- | --- | --- | --- |
| Gastric ulcer (K25) | 384 | 318.67 | 42.7 | 57.3 |
| Duodenal ulcer (K26) | 206 | 268.24 | 56.3 | 43.7 |
| Unspecified (K27) | 10 | 420.00 | 10.0 | 90.0 |
| Gastrojejunal (K28) | 6 | 349.08 | 16.7 | 83.3 |

Higher mean rank corresponds to older age.

Kruskal–Wallis H = 16.077, p = 0.001.

Chi-square (df = 3) = 17.689, p = 0.001.
